# 784 Burn Injuries During Pregnancy

**DOI:** 10.1093/jbcr/irae036.325

**Published:** 2024-04-17

**Authors:** Jordan Jacobs, Elisabeth A Carter, Felicia Williams, Lori Chrisco, booker King

**Affiliations:** The University of North Carolina, Chapel Hill, NC; The University of North Carolina at Chapel Hill, Chapel Hill, NC; NC Jaycee Burn Center, Chapel Hill, NC; UNC Chapel Hill. NC, Chapel Hill, NC; The University of North Carolina, Chapel Hill, NC; The University of North Carolina at Chapel Hill, Chapel Hill, NC; NC Jaycee Burn Center, Chapel Hill, NC; UNC Chapel Hill. NC, Chapel Hill, NC; The University of North Carolina, Chapel Hill, NC; The University of North Carolina at Chapel Hill, Chapel Hill, NC; NC Jaycee Burn Center, Chapel Hill, NC; UNC Chapel Hill. NC, Chapel Hill, NC; The University of North Carolina, Chapel Hill, NC; The University of North Carolina at Chapel Hill, Chapel Hill, NC; NC Jaycee Burn Center, Chapel Hill, NC; UNC Chapel Hill. NC, Chapel Hill, NC; The University of North Carolina, Chapel Hill, NC; The University of North Carolina at Chapel Hill, Chapel Hill, NC; NC Jaycee Burn Center, Chapel Hill, NC; UNC Chapel Hill. NC, Chapel Hill, NC

## Abstract

**Introduction:**

Burn injury during pregnancy is uncommonly studied, but represents a potentially devastating public health crisis. There is the potential for multiple people injured and lives lost. The aim of this study was to review our institution’s experience with this rare subgroup and to isolate specific trends.

**Methods:**

A retrospective study of burn injuries in pregnant women, admitted from 2013-2023 to a single burn center, was conducted to determine outcomes of pregnant patients. Data on these patients were collected utilizing the burn registry and a manual chart review.

**Results:**

Forty patients were identified and stratified by age, weeks of gestation, mechanism of burn injury, TBSA, length of stay, ICU status, surgical intervention, maternal and fetal mortality, and substance use. The mean average age was 27.6 years, and patients were, on average, 20.8 weeks pregnant. The majority of the injuries being sustained were from scald burns (22), followed by flame (12), chemical (3), contact (2), and electrical (1), with one reported inhalation injury. TBSA ranged from 0-40%, with an average TBSA of 4.5%. Length of stay averaged 5.3 days, and 12 patients were admitted to the ICU, with a mean ICU length of stay of 4.3 days. The majority of patients did not receive any surgical intervention, but for those who did, they received either skin replacement (11), or skin substitute (4). During this time, there was one live birth, and no maternal or fetal deaths. Of those admitted, 12 (30%) tested positive for illegal substance use (22.5% marijuana, 7.5% cocaine), and 5 (12.5%) identified as a smoker.

**Conclusions:**

The high incidence of substance use in this population was a surprising finding and warrants further investigation. There is a need for a multi-center, retrospective study to better understand trends in this unique population, with a focus on substance use.

**Applicability of Research to Practice:**

Investigating substance use in this population will help us understand how to better treat these patients.

